# Intraoperative intra-aortic balloon pump improves 30-day outcomes of patients undergoing extensive coronary endarterectomy

**DOI:** 10.1186/s13019-020-01261-5

**Published:** 2020-08-20

**Authors:** Zhen Wu, Changcheng Liu, Ying Fang, Hua Wei, Chengxiong Gu

**Affiliations:** Department of Cardiac Surgery, Beijing Anzhen Hospital, Capital Medical University, Anzhen Street No.2 Chaoyang District, Beijing, 100029 China

**Keywords:** Diffuse coronary artery disease, Coronary endarterectomy, Intra-aortic balloon pump, Short-term outcomes

## Abstract

**Background:**

The efficacy of intra-aortic balloon pump (IABP) has been proven in high-risk patients undergoing coronary artery bypass grafting (CABG). However, data on the timing and benefits of IABP support in diffuse coronary artery disease after CABG combined with coronary endarterectomy (CE) remain scarce. This retrospective study assessed the effect of intraoperative or postoperative IABP on 30-day outcomes of off-pump CABG+CE.

**Methods:**

From January 2012 to December 2018, 546 patients undergone off-pump CABG+CE were divided into control group (*n* = 437) and IABP group (*n* = 109). Risk factors for 30-day outcomes were evaluated. Subgroup analysis from IABP group was conducted to identify the effect of timing IABP on 30-day outcomes.

**Results:**

CE on left anterior descending branch of coronary artery (LAD) (OR = 3.079, 95% CI 1.077–8.805, *P* = 0.036), CE with≥2 vessels (OR = 9.123, 95% CI 3.179–26.033, *P* < 0.001) and length of atherosclerotic plaque ≥3 cm (OR = 16.017, 95% CI 5.941–43.183, *P* < 0.001) were independent risk factors for postoperative acute myocardial infarction (AMI) and 30-day mortality. Comparing with intraoperative IABP support, postoperative IABP support (OR = 3.987, 95% CI1.194–13.317, *P* = 0.025) was closely associated with postoperative AMI and 30-day mortality.

**Conclusions:**

For patients undergone off-pump CABG and extensive CE (CE on LAD, CE ≥2 vessels and length of atherosclerotic plaque ≥3 cm), intraoperative IABP support may improve 30-day outcomes.

## Background

Coronary artery bypass grafting (CABG) and coronary endarterectomy (CE) have been the established surgical strategy for myocardial revascularization of diffuse coronary artery disease [1–3]. However, postoperative acute myocardial infarction (AMI) secondary to acute thrombosis of the endarterectomized artery is a life-threatening complication after CABG+CE despite of advancing in surgical techniques and aggressive antithrombotic therapy [4, 5]. The latest meta-analysis study indicated that CABG+CE significantly increased the risk of perioperative myocardial infarction (OR = 3.17, 95% CI 1.75–5.75) and the risk of all-cause mortality after 30 days of surgery (OR = 1.86, 95% CI 1.66–2.08), compared with CABG alone [6].

Therefore, reducing the risk of acute thrombosis is vital for patients undergone CABG+CE.

The IABP can increase coronary blood flow, decrease afterload and improve hemodynamic stability, has been the most widely used mechanical circulatory support device in cardiac surgery [7, 8].

The efficacy of IABP has been proven in high-risk patients with severe left main coronary disease, recent AMI, or drug-refractory angina needing to CABG [9, 10]. But it is unclear whether patients undergone CABG+CE benefit from IABP. Currently, very few studies have investigated the efficacy and timing of IABP use in CABG+CE. Therefore, in this retrospective study, we assessed the effect of intraoperative or postoperative IABP support on 30-day outcomes in patients undergone CABG+CE.

## Methods

### Patient population and grouping

Between January 2012 and December 2018, 726 patients with diffuse coronary artery disease undergone off-pump CABG+CE were included in this study. Exclusion criteria included (1) preoperative IABP application duo to poor hemodynamic condition, left main coronary stenosis > 70%, drug-refractory angina; (2) urgent or emergent CABG; (3) undergoing concurrent valvular or aortic surgery; (4) urgent switching from off-pump to on-pump CABG during intraoperation.

Referring to the exclusion criteria, a total of 546 patients were included in this retrospective study. According to whether or not IABP support, all patients were allocated to control group (without IABP support, *n* = 437), and IABP group (*n* = 109). Moreover, IABP group was divided into two subgroups: intraoperative IABP group (*n* = 48) and postoperative IABP group (*n* = 61) referring to the timing of IABP support.

### Off-pump CABG+CE

All patients underwent standard procedural protocol involving median sternotomy under general anesthesia and harvesting of the left internal mammary artery (LIMA) and saphenous vein (SV). Off-pump CABG was performed after systemic heparinization with activated clotting time (ACT) of > 300 s. The LIMA was always grafted to the left anterior descending coronary artery (LAD). The proximal anastomosis of SV graft to the ascending aorta was performed firstly. The distal anastomosis of SV was then performed from the diagonal coronary artery, followed by sequential grafting of circumflex coronary artery and right coronary artery. In some patients with indications, the bilateral internal mammary arteries were used with “Y” configuration graft [11].

The CE was performed by as follow principles: (1) coronary angiography indicating diffuse lesions with length > 2 cm, luminal diameter < 1 mm or chronic total occlusion in the main branch; (2) intraoperative inspection finding no suitable anastomotic location in the middle-distal segment of the target artery but with a wide blood supply area. The atherosclerotic plaque was removed by closed-CE with a limit arteriotomy based on the anastomotic size. The satisfactory standard for CE was that the end of the plaque from the distal end of the target artery was with a translucent and rat-tail shape, and blood outflowed via arteriotomy. And the open-CE with extended arteriotomy and patch angioplasty was performed for complete removing plaque, if the residual plaque was thought to remain in the distal target artery after closed-CE. The details of CE procedure referred to the previous study [12]. All patients received the dual antiplatelet therapy within 6–24 h postoperatively, until 12 months after surgery.

### IABP support

IABP was implanted via femoral artery. A 7 or 7.5 F balloon catheter (30- or 40-ml balloon depending on the height and weight of patients; Arrow, Datascope Corp, USA) was placed in the descending aorta and connected to a Datascope pump (Datascope, Oakland, N.J., USA). The right position of balloon catheter was identified by chest X-ray. Unless contraindicated, the IABP-supported patients received intravenous heparin maintaining ACT > 180 s. IABP application was considered if patients met the following criteria: (1) hemodynamic monitor showing mean arterial pressure < 60 mmHg, pulmonary artery wedge pressure > 20 mmHg and central venous pressure > 15 mmHg, or cardiac index < 1.5 L/m^2^/min despite of inotropic agent support; (2) two or more inotropic agents > 5 μg/kg/min to maintain hemodynamic stability; (3) cardiopulmonary resuscitation duo to sudden cardiac arrest or malignant ventricular arrhythmias. IABP support was discontinued when hemodynamic stability with minimal inotropic agent, or improvement of global wall contraction and left ventricular ejection fraction.

### End-points

The primary end-points were postoperative AMI and 30-day mortality. The postoperative AMI was defined by (1) postoperative cTnI more than 10 times the 99th percentile of the upper limit of normal reference value with normal preoperative cTnI valve; (2) ongoing evidences of myocardial ischemia including new pathology Q wave formation, coronary angiography confirmed the presence of new coronary or grafts occlusion, imaging evidence of new viable myocardium loss or local wall motion abnormalities consistent with ischemic etiology [13].

The secondary end-points included duration of mechanical ventilation, and duration of ICU and postoperative hospital stays.

### Statistical analysis

Statistical analysis was performed using an extensively admissive software program SAS software (version 9.4; SAS Institute Inc., Cary, NC, USA). Data was presented as means ± standard deviation (SD) for continuous variables, and as frequencies and percentages for categorical variables. The ANOVA test was used to address non-paired samples for the comparison of normally distributed parameters, and the Wilcoxon rank-sum test for the comparison of non-parametric variables. The Chi-squared test and Fisher’s exact test were applied for the comparison of categorical variables. The multivariate logistic regression analysis was performed to calculate odds ratio (OR) and 95% confidence interval (CI) for identifying the risk factors and impact of IABP timing for primary end-points of off-pump CABG+CE. Differences were considered statistically significant only when *p*-value was < 0.05.

## Results

### Baseline characteristics

A total of 546 patients with 77.29% male were studied. Comparing with the control group, patients in IABP group were companied of increased BMI (26.70 ± 2.58 vs 25.87 ± 2.76, *P* = 0.005). And the incidence of diabetes mellitus in IABP group was higher than control group (53.21% vs 40.96%, *P* = 0.021). The characteristic distribution of other demographics, cardiac function parameters, comorbidities in two groups were non-significant difference. The baseline data of patients were listed in Table 1.

**Table 1:** Baseline characteristics of patients undergoing off-pump CABG+CE

variables	Control group (*n* = 437)	IABP group (*n* = 109)	*P* valve
Age (years)	61.25 ± 8.52	60.66 ± 8.53	0.518
Gender (males; %)	340 (77.80)	82 (75.23)	0.566
BMI (kg/m2)	25.87 ± 2.76	26.70 ± 2.58	0.005
Smoking (%)	162 (37.16)	33 (30.28)	0.185
Hypertension (%)	272 (62.24)	73 (66.97)	0.360
Diabetes mellitus (%)	179 (40.96)	58 (53.21)	0.021
Prior stroke (%)	64 (14.64)	10 (9.17)	0.135
PVD	152 (34.78)	39 (35.78)	0.845
LVEF	61.02 ± 7.38	60.47 ± 8.74	0.546
LVEDD	49.92 ± 5.05	50.72 ± 5.92	0.196
NYHA class III or IV	12 (2.74)	4 (3.67)	0.538

*BMI* body mass index, *CABG* coronary artery bypass grafting, *CE* coronary endarterectomy, *PVD* peripheral vascular diseases, *LVEF* left ventricular ejection fraction, *LVEDD* left ventricular end diastolic diameter

### Surgical characteristics and 30-day outcomes

Comparing with the control group, the proportion of CE on LAD, CE with ≥2 vessels and length of atherosclerotic plaque ≥3 cm were higher in IABP group, *P*<0.001. Moreover, patients in IABP group received longer duration of mechanical ventilation, ICU stay and postoperative hospital stay with higher incidence of postoperative AMI and 30-mortality than patients in control group, *P*<0.001. The details were showed in Table 2.

**Table 2:** Surgical parameters and in-hospital outcomes of two groups

variables	Control group (*n* = 437)	IABP group (*n* = 109)	*P* valve
Number of graft anastomosis	3.86 ± 0.54	3.89 ± 0.55	0.605
LIMA-LAD anastomosis (%)	397 (90.85)	97 (88.99)	0.555
CE on LAD (%)	42 (9.61)	46 (42.20)	< 0.001
CE ≥2 vessels (%)	57 (13.04)	58 (53.21)	< 0.001
length of atherosclerotic plaque ≥3 cm (%)	43 (9.84)	72 (66.06)	< 0.001
Postoperative AMI	16 (3.67)	21 (19.27)	< 0.001
30-day mortality	4 (0.92)	11 (10.09)	< 0.001
Duration of mechanical ventilation (hours)	24.90 ± 89.29	55.26 ± 85.89	< 0.001
Length of ICU stay (days)	1.57 ± 4.57	3.02 ± 2.99	< 0.001
Length of postoperative hospital stays (days)	7.99 ± 4.87	11.15 ± 7.91	< 0.001

*CE* coronary endarterectomy, *ICU* intensive care unit, *LAD* left anterior descending branch of coronary arterym, *LIMA* left internal mammary artery

### Risk factors for primary end-points

**Fig. 1:**
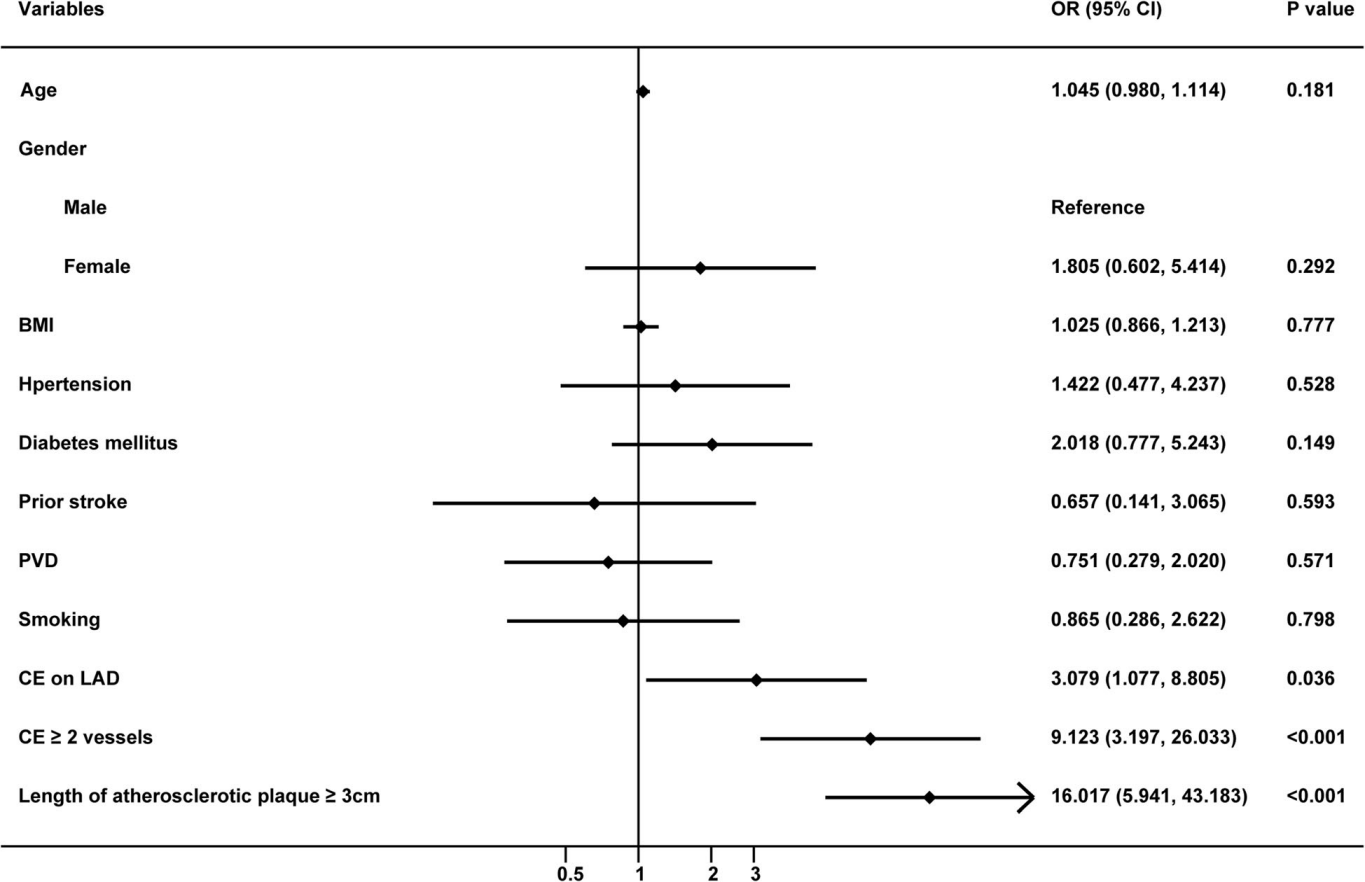
Logistic regression analysis of risk factors for postoperative AMI and 30-day mortality after off-pump CABG+CE. AMI: acute myocardial infarction; CABG: coronary artery bypass grafting; CE: coronary endarterectomy

The multivariate logistic regression analysis (Fig. 1) indicated that CE on LAD (OR = 3.079, 95% CI 1.077–8.805, *P* = 0.036), CE with ≥2 vessels (OR = 9.123, 95% CI 3.179–26.033, *P* < 0.001) and length of atherosclerotic plaque ≥3 cm (OR = 16.017, 95% CI 5.941–43.183, *P* < 0.001) were independent risk factors for postoperative AMI and 30-day mortality.

In terms of timing of IABP application, patients in the postoperative IABP group had a higher proportion of CE on LAD, CE with ≥2 vessels and length of atherosclerotic plaque ≥3 cm than that of intraoperative IABP group, *P* < 0.05. The intraoperative IABP support significantly reduced the incidence of postoperative AMI (10.42% vs 26.23%, *P* = 0.038) than postoperative IABP support. But IABP-related complications including low limb ischemia requiring surgery and severe bleeding were no significantly different between intra-, and post-operative IABP support. The details of subgroup analysis were showed in Table 3.

**Table 3:** Surgical parameters and in-hospital outcomes of patients with different phase of IABP support

variables	Intraoperative IABP group (*n* = 48)	Postoperative IABP group (*n* = 61)	*P* valve
Number of graft anastomosis	3.98 ± 0.50	3.82 ± 0.58	0.132
LIMA-LAD anastomosis (%)	44 (91.67)	53 (86.88)	0.429
CE on LAD (%)	15 (31.25)	31 (50.82)	0.040
CE ≥2 vessels (%)	13 (27.08)	45 (73.77)	< 0.001
length of atherosclerotic plaque ≥3 cm (%)	26 (54.17)	46 (75.41)	0.020
Postoperative AMI	5 (10.42)	16 (26.23)	0.038
30-day mortality	2 (4.17)	9 (14.75)	0.108
Duration of mechanical ventilation (hours)	54.67 ± 78.69	55.72 ± 92.77	0.895
Length of ICU stay (days)	2.88 ± 2.86	3.13 ± 3.15	0.667
Length of postoperative hospital stays (days)	11.07 ± 5.79	11.21 ± 9.43	0.925
IABP complications			0.445
Lower limb ischemia requiring surgery	1	0	
Severe bleeding	0	0	

*CE* coronary endarterectomy, *IABP* intra-aortic balloon pump, *ICU* intensive care unit, *LAD* left anterior descending branch of coronary artery, *LIMA* left internal mammary artery

**Fig. 2:**
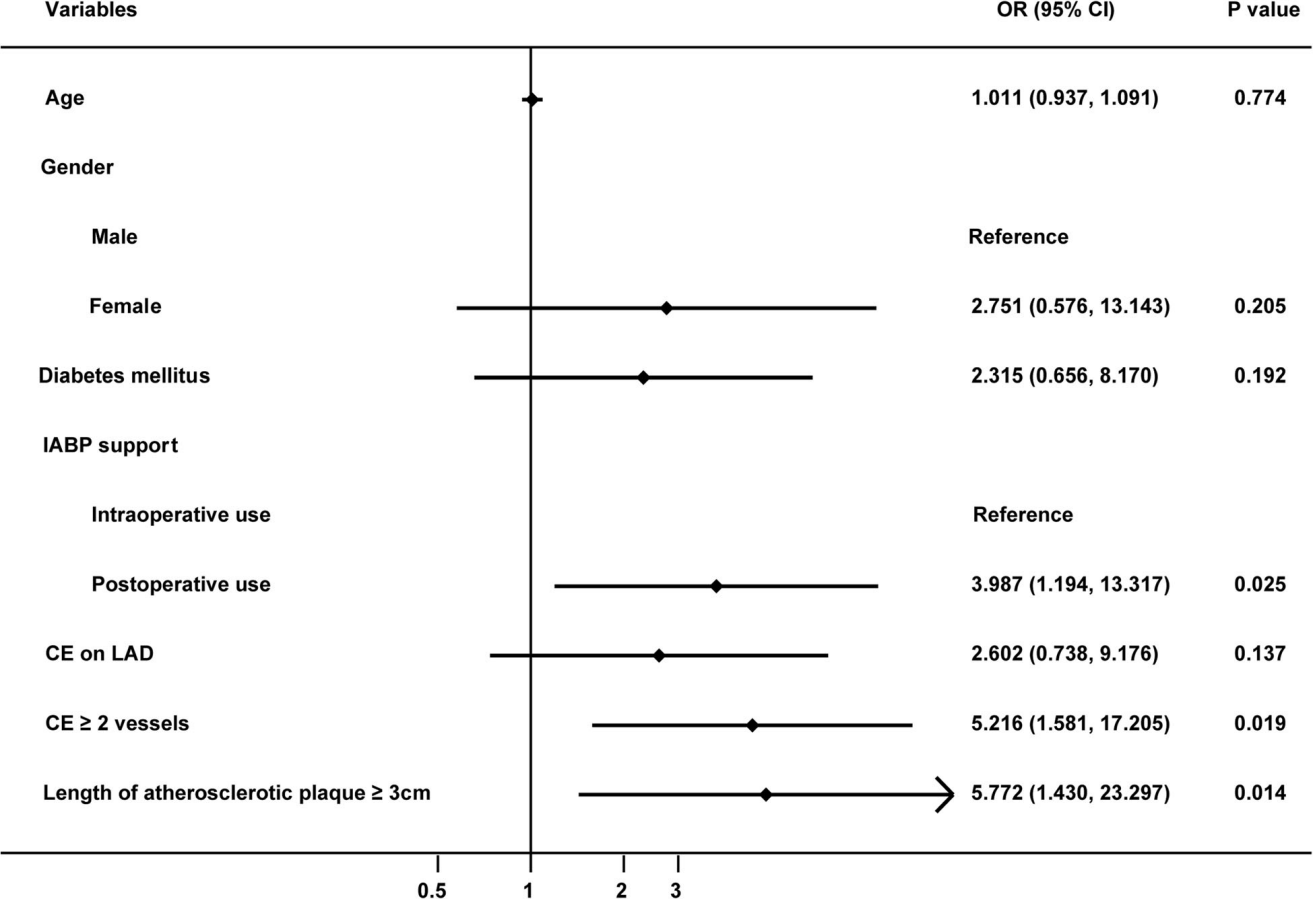
Logistic regression analysis of risk factors for postoperative AMI and 30-day mortality in the IABP group. AMI: acute myocardial infarction; IABP: intra-aortic balloon pump

The multivariate logistic regression analysis in subgroup (Fig. 2) indicated that CE with ≥2 vessels (OR = 5.216, 95% CI 1.581–17.205, *P* = 0.019) and length of atherosclerotic plaque ≥3 cm (OR = 5.772, 95% CI1.430–23.297, *P* = 0.014) remarkably increased risk of postoperative AMI and 30-day mortality. And postoperative IABP support (OR = 3.987, 95% CI1.194–13.317, *P* = 0.025) was closely associated with postoperative AMI and 30-day mortality, implicating that intraoperative IABP support may be a protective factor for primary end-points.

## Discussion

This study firstly investigated the effect of timing of IABP support on 30-day outcomes in patients undergone off-pump CABG+CE. The main findings included that comparing with postoperative IABP support, intra-operative IABP support significantly reduced postoperative AMI in patients received extensive CE (CE with ≥2 vessels and length of atherosclerotic plaque ≥3 cm).

Complete revascularization is one of the main advantages of CABG. For diffuse coronary disease, residual stenosis of coronary artery increases the risk of postoperative AMI and mortality, and the need of revascularization duo to late recurrence of angina pectoris [14]. Therefore, CE seems to be a mandatory surgical option in this complex coronary lesion, particularly bypass grafting on the LAD and right coronary arteries.

CE mainly affected the early outcomes after surgery. Many studies reported that the incidence of AMI and operative mortality after CABG+CE were 3.8–13.9% and 2.0–6.5%, respectively, and they were higher than CABG alone [2, 12, 15]. In despite of increasing early risks of morbidity and mortality after CE, the long-term outcomes of CABG+CE were not inferior to the CABG alone. A propensity matched cohorts study (1074 patients) comparing the long-term outcomes in patients undergone CABG with or without CE suggested no significant difference in terms of survival rate (65.8% versus 70.7%) and coronary reintervention (11.6% versus 12.7%) during10 years follow-up [16]. Furthermore, Schwann et al. analyzed the late graft patency of 288 patients undergone CABG+CE, and found that the graft failure rate after CABG+CE vs CABG alone was no significant difference during 1-year follow-up [17].

The main reason causing poor early postoperative outcomes after CABG+CE is acute thrombosis duo to the damage and attenuation of endothelium in the target arteries. Maintaining the structural and functional integrity of coronary endothelium is pivotal to inhibit platelet adhesion and aggregation, and thrombosis pathway, whereas the CE may destroy the structural and functional integrity of coronary endothelium, which increases the risk of acute thrombosis [18]. In addition, the widespread lesion up to the distal part of the coronary artery does not always allow complete revascularization of myocardium territories, which also contribute to the high occurrence of AMI after CE.

Although advancing techniques in CABG+CE and aggressive postoperative antiplatelet and anticoagulant therapy, a recent meta-analysis of 54,440 patients reported that the incidence of postoperative AMI and operative mortality were up to 5.2 and 4.3% after CABG+ CE, respectively [19]. So, early postoperative acute thrombosis should be closely attention. On the basis of optimal antithrombotic therapy, increasing coronary blood perfusion maybe reduce the risk of acute thrombosis after CE.

IABP is low-cost, relative simplicity to use and easy to management in the clinical settings, has been the most common used mechanical circulation assist device in patients requiring hemodynamic support [20]. In theory, IABP can support the heart indirectly by the following hemodynamic effects: (1) increasing diastolic aortic pressure with subsequent enhancement in diastolic blood flow resulting in better perfusion of coronary artery; (2) reducing systolic aortic pressure by decreasing the afterload, subsequently decreasing left ventricle wall stress and then reducing the myocardial oxygen consumption. Stefanadis et al. demonstrated that IABP could cause a 30% increase in aortic distensibility and thereby reducing the aortic stiffness constant, which resulted in 24% increase in cardiac index and a 31% reduction in myocardial oxygen demand [21].

Furthermore, intraoperative IABP support maybe reduce the risk of acute thrombosis after CE. Possible mechanisms contributing to reduce acute thrombosis with intraoperative IABP support are as follows: (1) improving coronary perfusion increases coronary blood flow velocity and thereby reducing the risk of blood stasis which is one of the three major causes of thrombosis; (2) increasing coronary blood pressure augments vascular stretch induced endothelial-derived nitric oxide release during the diastolic phase, and nitric oxide can result in diastolic arteriolar vasodilation and inhibit platelet aggregation reducing possibility of thrombosis [22]; (3) during IABP support, ACT is always maintained more than 180 s by intravenous heparin, which contribute to keep the stabile anticoagulation condition in circulation. Above circulation support advantages and hemodynamic benefits of IABP partially explained our study results that intraoperative IABP support remarkably reduced postoperative AMI than postoperative IABP support in patients with CABG+ CE.

However, some patients in postoperative IABP group maybe suffer from acute thrombosis in the CE-related coronary arteries before IABP implantation. Kimura et al. [23] found that IABP did not increase significantly blood perfusion in post-stenotic coronary arteries territory unless eliminating stenosis via thrombolysis or percutaneous coronary intervention, which may explain why postoperative IABP did not reduce postoperative AMI after CABG+CE.

In addition, the surgical techniques may affect the early outcomes of CABG+CE. Surgical techniques treating diffuse coronary disease include off-pump CABG and on-pump CABG for myocardial revascularization, and open-CE and closed-CE for removing atherosclerotic plaques. A systematic review study reported that no statistical difference in 30-day mortality was found between open-CE and closed-CE [24]. And Lee et al. [3] also demonstrated that no statistical difference in operative mortality was found between the on-pump CABG +CE and off-pump CABG+CE groups or between open-CE and closed-CE. We mainly applied off-pump CABG and closed-CE to treat diffuse coronary disease in this study. Regardless of surgical techniques, thorough removing plaques at the distal part of target artery and complete revascularization were the key to improve surgical outcomes.

Very limited evidence is available to evaluate the impact of the prophylactic IABP use in patients with CABG+CE. In our series, multivariate regression analysis showed that extensive CE (CE on LAD, CE ≥2 vessels and length of atherosclerotic plaque ≥3 cm) increased remarkably postoperative AMI and 30-mortality. Therefore, for patients undergoing extensive CE, intraoperative prophylactic IABP use maybe reduce the risk of postoperative AMI and then improve early outcomes after CABG+CE.

Furthermore, IABP is a very safe circulation support device. The Benchmark Registry reports an incidence of 2.6% for major complications related to IABP use (severe limb ischemia, severe bleeding, balloon leak, or death due to IABP insertion), and only 0.05% of in-hospital mortality was directly attributable to IABP [25]. In our study, the IABP-related complications were very low without 30-day mortality caused by IABP directly.

Several limitations in this study should be considered. First, the study was susceptible to inherent bias from retrospective nature. Second, the cohort was relatively small because the enrolled patients belonged to the high-selective subgroup with severe diffuse coronary disease and needing IABP support after CABG and extensive CE. Third, in subgroup analysis, compared with intraoperative IABP group, the patients in postoperative IABP group underwent more extensive CE with higher risk of AMI, which may be one of confounding factors. A larger sized, CE degree-matching study should be conducted to further evaluate the efficacy of prophylactic IABP support during intraoperation in patients received extensive CE. Fourth, we mainly applied off-pump CABG + closed CE. So, it was not clear to whether patients undergoing extensive CE benefited from intraoperative IABP support after on-pump CABG + open CE.

## Conclusions

The extensive CE including CE on LAD, CE ≥2 vessels and length of atherosclerotic plaque ≥3 cm remarkably increased postoperative AMI and 30-mortality. Intraoperative IABP support remarkably reduced postoperative AMI and 30-mortality than postoperative IABP support in patients undergone off-pump CABG + CE.


**Additional file 1**

## Data Availability

The datasets used are available from the corresponding author on reasonable request.

## Abbreviations

ACT: Activated clotting time
AMI: Acute myocardial infarction
BMI: Body mass index
CABG: Coronary artery bypass grafting
CE: Coronary endarterectomy
IABP: Intra-aortic balloon pump
LAD: Left anterior descending branch of coronary artery
LIMA: Left internal mammary artery
SV: Saphenous vein
